# Verification, analytical validation, and clinical validation (V3): the foundation of determining fit-for-purpose for Biometric Monitoring Technologies (BioMeTs)

**DOI:** 10.1038/s41746-020-0260-4

**Published:** 2020-04-14

**Authors:** Jennifer C. Goldsack, Andrea Coravos, Jessie P. Bakker, Brinnae Bent, Ariel V. Dowling, Cheryl Fitzer-Attas, Alan Godfrey, Job G. Godino, Ninad Gujar, Elena Izmailova, Christine Manta, Barry Peterson, Benjamin Vandendriessche, William A. Wood, Ke Will Wang, Jessilyn Dunn

**Affiliations:** 1 Digital Medicine Society (DiMe), Boston, MA USA; 2 Elektra Labs, Boston, MA USA; 3 Harvard-MIT Center for Regulatory Science, Boston, MA USA; 4 Philips, Monroeville, PA USA; 5 Biomedical Engineering Department, Duke University, Durham, NC USA; 6 Takeda Pharmaceuticals, Cambridge, MA USA; 7 ClinMed LLC, Dayton, NJ USA; 8 Computer and Information Sciences Department, Northumbria University, Newcastle-upon-Tyne, UK; 9 Center for Wireless and Population Health Systems, University of California, San Diego, CA USA; 10 Samsung Neurologica, Danvers, MA USA; 11 Curis Advisors, Cambridge, MA USA; 12 Koneksa Health, New York, USA; 13 Independent Consultant, Charlotte, NC USA; 14 Byteflies, Antwerp, Belgium; 15 Department of Electrical, Computer and Systems Engineering, Case Western Reserve University, Cleveland, OH USA; 16 Department of Medicine, University of North Carolina at Chapel Hill; Lineberger Comprehensive Cancer Center, Chapel Hill, NC USA; 17 Department of Biostatistics & Bioinformatics, Duke University, Durham, NC USA

**Keywords:** Scientific community, Research data

## Abstract

Digital medicine is an interdisciplinary field, drawing together stakeholders with expertize in engineering, manufacturing, clinical science, data science, biostatistics, regulatory science, ethics, patient advocacy, and healthcare policy, to name a few. Although this diversity is undoubtedly valuable, it can lead to confusion regarding terminology and best practices. There are many instances, as we detail in this paper, where a single term is used by different groups to mean different things, as well as cases where multiple terms are used to describe essentially the same concept. Our intent is to clarify core terminology and best practices for the evaluation of Biometric Monitoring Technologies (BioMeTs), without unnecessarily introducing new terms. We focus on the evaluation of BioMeTs as fit-for-purpose for use in clinical trials. However, our intent is for this framework to be instructional to all users of digital measurement tools, regardless of setting or intended use. We propose and describe a three-component framework intended to provide a foundational evaluation framework for BioMeTs. This framework includes (1) verification, (2) analytical validation, and (3) clinical validation. We aim for this common vocabulary to enable more effective communication and collaboration, generate a common and meaningful evidence base for BioMeTs, and improve the accessibility of the digital medicine field.

## Introduction

Digital medicine describes a field concerned with the use of technologies as tools for measurement and intervention in the service of human health. Digital medicine products are driven by high-quality hardware, firmware, and software that support the practice of medicine broadly, including treatment, intervention, and disease prevention, as well as health monitoring and promotion for individuals and across populations^1^.

Isolated silos of knowledge exist within the engineering, technology, data science, regulatory, and clinical communities that are critical to the development and appropriate deployment of digital medicine products. Currently, terminology, approaches, and evidentiary standards are not aligned across these communities, slowing the advancement of digital medicine for improved health, healthcare, and health economics. Consensus approaches are needed to evaluate the quality of digital medicine products, including their clinical utility, cybersecurity risks, user experience, and data rights and governance for ‘digital specimen’ collection^2^.

In this work, we refer to a specific type of digital medicine product that we call Biometric Monitoring Technologies, or BioMeTs. BioMeTs are connected digital medicine products that process data captured by mobile sensors using algorithms to generate measures of behavioral and/or physiological function. This includes novel measures and indices of characteristics for which we may not yet understand the underlying biological processes. BioMeTs, like other digital medicine products, should be characterized by a body of evidence to support their quality, safety, and effectiveness^3^. However, the rapid rise in the development of and demand for BioMeTs to support the practice of medicine has left in its wake a knowledge gap regarding how to develop and evaluate this body of evidence systematically^4^. If not addressed, there is potential for misinterpretation of data resulting in misleading clinical trials and possibly patient harm.

What are the necessary steps to determine whether a metric derived from a BioMeT is trustworthy, and by extension, whether that BioMeT is fit-for-purpose? We begin by exploring and adapting applicable concepts from other standards in related fields. Digital medicine is an interdisciplinary and rapidly evolving field. The Biomarkers, EndpointS, and other Tools (B.E.S.T) framework emphasizes that “effective, unambiguous communication is essential for efficient translation of promising scientific discoveries into approved medical products”^5^. Siloed and non-standardized practices will slow down innovation and impede collaboration across domains.

In this manuscript, we develop an evaluation framework for BioMeTs intended for healthcare applications. This framework includes verification, analytical validation, and clinical validation (V3). We propose definitions intended to bridge disciplinary divides and describe how these processes provide foundational evidence demonstrating the quality and clinical utility of BioMeTs as digital medicine products.

## Language matters and should be used intentionally

Establishing a common language to describe evaluation standards for BioMeTs is critical to streamline trustworthy product development and regulatory oversight. In this paper, we avoid using the term “device” because we anticipate that there is a potential regulatory context for the V3 framework. We want to avoid confounding the V3 terminology with existing FDA Terms of Art (e.g., “device”). Instead, we intentionally discuss digital medicine products, and specifically BioMeTs. We refer the reader to Coravos et al for more background on regulatory considerations^3^. In addition, in this manuscript we use the term “algorithm” to describe a range of data manipulation processes embedded in firmware and software, including but not limited to signal processing, data compression and decompression, artificial intelligence, and machine learning.

We also avoid using the term “feasibility study.” These studies can be purposed to evaluate the feasibility of a number of performance questions and so “feasibility study” in isolation is a meaningless term. We use the term “gold standard” in quotations because it often refers to entrenched and commonly used measurement standards that are considered sub-optimal. “Gold standards” should be considered as nothing more than the best available measurement per consensus, against which the accuracy of other measurements of similar purposes may be judged^6^.

In this paper, we use the term “data supply chain” to describe data flow and data provenance for information generated from hardware, sensors, software, and algorithms.

## Why V3?

Two terms, verification and validation, have been used for decades to describe critical components of successful quality management systems. The ISO 9000 family of quality management system standards, first published in 1987, have specific standards and definitions related to design verification and validation^7^. These ISO 9000 standards are generic and can be applied to any type of organization; as such, many industries have adapted these standards to their specific needs. For example, ISO 13485 specifies quality management system requirements related to design verification and validation for organizations that provide medical devices and related services^8^.

In the most basic sense, a BioMeT combines software and hardware for medical or health applications. The software, hardware, and regulatory parent industries have long histories of verification and validation as part of their quality management systems. Software and hardware verification and validation are guided by the IEEE Standard for System, Software, and Hardware Verification and Validation (IEEE 1012-2016), which lays out specific requirements that must be met in order to comply with the standard^9^. The FDA also describes verification and validation processes required for software and hardware products that are submitted for their approval^10,11^.

Traditional validation for software and hardware products confirms that the end product accurately measures what it claims to measure. However, BioMeT-derived measures from digital tools must also be clinically useful to a defined population. As such, we have split validation into analytical validation and clinical validation, similar to the framework used in the development of wet biomarkers and described in the BEST (Biomarkers, EndpointS, and other Tools) resource developed by the FDA-NIH Biomarkers working group^5^.

The three-component V3 framework is novel and intentionally combines well established practices from both software and clinical development. The definitions for V3 were derived from guidance documents, historical, and current frameworks ranging from 2002 to 2018. Each document referenced focuses on the particular audience for its associated organization(s), including system developers and suppliers, pharmaceutical industry sponsors, and regulators (Table 1). The context of the definitions provided for V3 vary greatly, highlighting that language and processes are often generated and used within disciplinary silos. Although some commonalities exist, the comparisons are confusing at best (Supplementary Table 1). These communities also lack a standard language to describe the data supply chain for information generated from the hardware, sensors, software, and algorithms.

**Table 1:** Existing definitions of V&V or similar concepts in a selection of reference and guidance documents from disciplines contributing to digital medicine.

Source of guidance document	IEEE (2016)^43^	BEST (2018)^5^	CTTI (2018)^14^	SaMD (2017)^17^	FDA (2002)^42^	NASEM (2017)^44^
Intended audience for document	System, software, and hardware suppliers, acquirers, developers, maintainers, V&V practitioners, operators, users, and managers in both the supplier and acquirer organizations	Broad stakeholder group (e.g., regulators, medical product manufacturers, patients)	Biotech & pharmaceutical sponsors, contract research organizations (CROs) and outsourced electronic service vendors, such as mobile technology manufacturers	International Regulatory Community	• Persons subject to the medical device quality system regulation • Persons responsible for the design, development, or production of medical device software • Persons responsible for the design, development, production, or procurement of automated tools used for the design, development, or manufacture of medical devices or software tools used to implement the quality system itself • FDA investigators • FDA compliance officers • FDA scientific reviewers	Multi-stakeholder community engaged in genetic and diagnostic testing
Are terms V&V defined?						
Verification	Yes	No	Yes	In prerequisite documents	Yes	No
Validation	Yes (does not split out analytical vs. clinical)	Yes (splits out analytical vs. clinical)	Yes (refers to analytical validation only)	Yes (splits out analytical vs. clinical validation; also includes clinical association/scientific validity)	Yes	Yes (splits out analytic vs clinical validation; also includes clinical utility)
What’s the context of V&V definitions?	Provides standards for V&V of software, hardware, and systems	Gives definitions & examples of biomarkers and surrogate endpoints; additional focus on COA (clinical outcome assessment)— specific validation (e.g., construct, content & criterion)	Advancing the use of mobile technologies for data capture & improved clinical trials	Describes an approach for planning the process for clinical evaluation of a SaMD (software with a medical purpose)	Describes how provisions of the medical device quality system regulation apply to software and the FDA’s approach to evaluating a software validation system	Developed in the context of providing recommendations to advance the development of an adequate evidence base for genetic tests to improve patient care and treatment. Uses the CDC’s ACCE model of 44 targeted questions
What’s missing from V&V definitions?	Data processing algorithm Clinical validation	Data processing algorithm	Relationship of digital metric to a meaningful clinical state or experience Clinical care applications	Hardware (decoupled from software) View of full data- supply chain	Hardware (decoupled from software) View of full data supply chain Clinical validation	Sensor hardware

Given (1) the historical context for the terms verification and validation in software and hardware standards, regulations, and guidances, and (2) the separated concepts of analytical and clinical validation in wet biomarkers development, this paper seeks to adapt existing terminology and evaluation frameworks for use in BioMeTs. In this new era of digital medicine, we suggest a broad interdisciplinary approach and a common lexicon containing consensus definitions across disciplines for these important terms.

## Moving from current siloed practices to one universal best practice

**Fig. 1:**
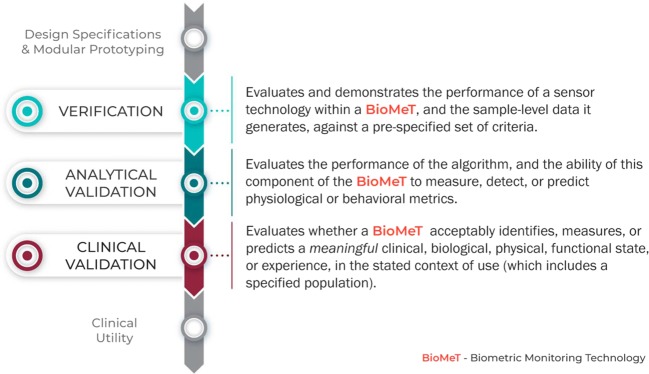
The stages of V3 for a BioMeT: Verification, analytical validation, and clinical validation of BioMeTs is a multi-step process. The stages of V3 for a BioMeT.

Evaluation of BioMeTs should be a multi-step process that includes relevant expertize at each stage, as well as interdisciplinary collaboration throughout. We propose V3, a three-component framework for the evaluation of BioMeTs in digital medicine (Fig. 1):

1. Verification of BioMeTs entails a systematic evaluation by hardware manufacturers. At this step, sample-level sensor outputs are evaluated. This stage occurs computationally in silico and at the bench in vitro.
2. Analytical validation occurs at the intersection of engineering and clinical expertize. This step translates the evaluation procedure for BioMeTs from the bench to in vivo. Data processing algorithms that convert sample-level sensor measurements into physiological metrics are evaluated. This step is usually performed by the entity that created the algorithm, either the vendor or the clinical trial sponsor.
3. Clinical validation is typically performed by a clinical trial sponsor to facilitate the development of a new medical product^12^. The goal of clinical validation is to demonstrate that the BioMeT acceptably identifies, measures, or predicts the clinical, biological, physical, functional state, or experience in the defined context of use (which includes the definition of the population). This step is generally performed on cohorts of patients with and without the phenotype of interest.

V3 must be conducted as part of a comprehensive BioMeT evaluation. However, although V3 processes are foundational, they are not the only evaluation steps. The concept we propose here is analogous to FDA’s Bioanalytical Method Validation Guidance for Industry^13^, which captures key elements necessary for successful validation of pharmacokinetic and wet laboratory biomarkers in the context of drug development clinical trials though there are some fundamental differences due to the nature of data collection tools and methods.

Clinical utility, which evaluates whether using the BioMeT will lead to improved health outcomes or provide useful information about diagnosis, treatment, management, or prevention of a disease is also necessary to determine fit-for-purpose^5^. To evaluate the clinical utility of a digital tool, the range of potential benefits and risks to individuals and populations must be considered, along with the relevance and usefulness of the digital product to individuals (e.g., adherence to using the technology, user experience, and battery life). Clinical utility is typically evaluated by a process of usability and user experience testing. A BioMeT may perform well under V3, but is useless if it cannot be used appropriately by the target population in the anticipated setting. However, usability, and user experience are outside of the scope of the proposed V3 framework. Other criteria, such as cost, accessibility, compatibility, burden and ease of use, failure rates, and manufacturers’ terms of use and or customer service, are also critical to determining fit-for-purpose. These are described in more detail by the Clinical Trials Transformation Initiative (CTTI)^14^.

## How does V3 for BioMeTs fit within the current regulatory landscape?

In the United States, regulators evaluate the claim(s) a manufacturer makes for a product, rather than the product’s capabilities. In other words, a product may be categorized as a regulated “device” or “non-device” purely through a change in the manufacturer’s description of the product with no change to its functionality (e.g., no change to the hardware, firmware, or software).

The setting in which a BioMeT is used can also shift the regulatory framework. For instance, a wearable used in a clinical trial to support a drug application (e.g., to digitally collect an endpoint like heart rate) would not necessarily be considered a “device”. However, the exact same product sold in the post-market setting claiming to diagnose a condition like atrial fibrillation, would be a device under the current paradigm.

Recognizing recent shifts in the technology landscape, the US Congress signed the 21st Century Cures Act (Cures Act)^15^ into law on 13 December 2016, which amended the definition of “device” in the Food, Drug and Cosmetic Act to include software-based products. As a result, the FDA has been generating new guidance documents, updating policies, and considering better approaches to regulate software-driven products^16^. One novel approach has been to decouple the system into separate hardware and software components. For instance, the International Medical Device Regulators Forum defined ‘Software as a Medical Device (SaMD)’ as a software that performs independently of medical device hardware and that is intended to be used for medical purposes^17^. Importantly, this regulatory construct means that software (including algorithms), which lack a hardware component can be considered a “device” and thus, regulated by the FDA. For example, in 2018 two mobile applications that use either electrocardiogram (ECG) or photoplethymography data to generate “Irregular Rhythm Notifications” were granted De Novo clearance by the FDA^18,19^.

## Verification

The verification process evaluates the capture and transference of a sensor-generated signal into collected data. Verification demonstrates that a sensor technology meets a set of design specifications, ensuring that (A) the sensors it contains are capturing analog data appropriately, and (B) the firmware that modifies the captured data are generating appropriate output data. In lay terms, the process of verification protects against the risk of ‘garbage in, garbage out’ when making digital measurements of behavioral or physiologic functions. BioMeTs include sensors that sample a physical construct; for example, acceleration, voltage, capacitance, or light. Verification is a bench evaluation that demonstrates that sensor technologies are capturing data with a minimum defined accuracy and precision when compared against a ground-truth reference standard, consistently over time (intra-sensor comparison) and uniformly across multiple sensors (inter-sensor comparison). The choice of reference standard depends on the physical construct captured. For example, verification of an accelerometer would involve placing the sensor on a shaking bench with known acceleration, and using these data to calculate accuracy, precision, consistency, and uniformity. In all of these processes, the evaluation criteria and thresholds should be defined prior to initiating the evaluation tests in order to determine whether the pre-specified acceptance criteria have been met.

## The data supply chain

**Fig. 2:**
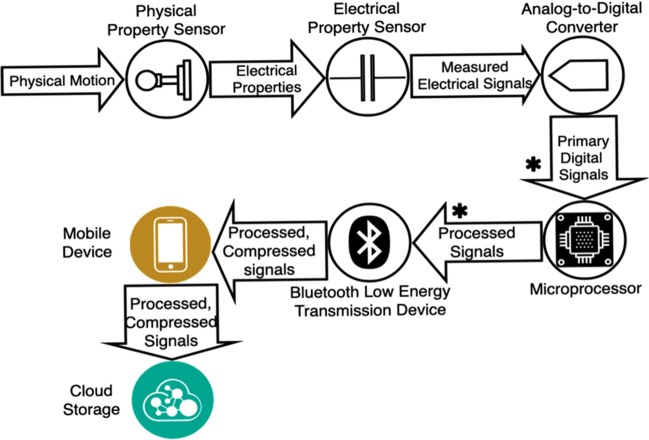
The “Raw” data dilemma: defining sample-level data in the data supply chain in a uniaxial MEMS accelerometer. Acceleration results in physical motion of the equivalence of a spring and proof mass, which in turn results in changes of electrical properties that can be captured by electrical property sensors. Electrical signals are then converted from analog to digital signals and stored and transmitted via the microprocessor on a wristband or mobile device. Through BLE, data are then processed and compressed multiple times for transmission and storage through mobile devices or cloud storage. This figure summarizes the steps of data collection and manipulation into a daily step count metric and illustrates that “raw” data could refer to different stages of the data collection and manipulation process and have different meanings. For more details of the data types and technologies involved in each step, please refer to Supplementary Table 2. Here, two arrows are highlighted with asterisks, which signify steps in the data supply chain where the “raw data dilemma” usually occurs. What is defined and clarified as “sample-level data” are the primary and processed digital signals marked by asterisks.

All digital measurements reported by BioMeTs are derived through a data supply chain, which includes hardware, firmware, and software components. For example, the accelerometer is a basic micro-electro-mechanical system frequently found in BioMeTs. Mechanical motion of a damped mass or cantilever in the accelerometer generates physical displacement information that can be translated through a series of data manipulations into a daily step count metric (Fig. 2; Supplementary Table 2). Each of these steps along the data supply chain has to be verified before the resulting measurement can be validated in a given population under specified conditions.

The term “raw data” is often used to describe data existing in an early stage of the data supply chain. Because the data supply chains vary across BioMeTs, the definition of “raw” is often inconsistent across different technologies. Here, we define the term sample-level data as a construct that holds clear and consistent meaning across all BioMeTs. All sensors output data at the sample level (for example, a 50 Hz accelerometer signal or a 250 Hz ECG signal); these data are sometimes accessible to all users and sometimes only accessible to the sensor manufacturers. We refer to this sensor output data as *d* and that data are reported in the International System of Units (SI). Although signal processing methods may have been applied to this data (e.g., downsampling, filtering, interpolation, smoothing, etc.), the data are still considered “raw” because it is a direct representation of the original analog signal produced by the sensor. These are the data that must undergo verification. Unfortunately, this sample-level data are often inaccessible to third parties using those technologies. This may be owing to limitations on storage space or battery life during transmission of high frequency data or it may be due to the risk of a third party reverse-engineering proprietary algorithms developed by the BioMeT manufacturer. In these situations, only the BioMeT manufacturer can complete verification of the sample-level data.

In summary, verification occurs at the bench prior to validation of the BioMeT in human subjects. Verified sample-level data generated from the sensor technology becomes the input data for algorithms that process that data into physiologically meaningful metrics (described further in analytical validation, below). Therefore, verification serves as a critical quality control step in the data supply chain to ensure that the sample-level data meet pre-specified acceptance criteria before the data are used further.

Table 2 summarizes the process of verification.

**Table 2:** Summary of verification.

Who?	Engineers, data & computer scientists
What?	Generation and preliminary processing of sample-level data
When?	Prior to testing the technology in human subjects
Where?	At the bench
Why?	To evaluate the performance of a sensor technology (1) against pre-specified criteria and (2) to demonstrate that the sample-level data generated is correct within the limits of the pre-specified conditions.

## How can we reconcile the process of verifying sensor technologies in digital medicine with approaches more familiar to other disciplines?

In both engineering and medicine, the goal of verification is to document that a specific device performs to intended specifications, but the details of the process vary with the sensor technology^20^. Verification itself is not defined by a fixed standard applied across all tools—rather, it is a declaration of performance with respect to a pre-specified performance goal. That performance goal is usually established by the manufacturer based on the intended use of the technology or by community standards for more common technologies, and can be precisely defined in terms that are easily testable. For example, an accelerometer’s intended performance circumstances may include the range of accelerations for which the accuracy exceeds 95% as well as the environmental and contextual conditions (e.g., temperature, humidity, battery level) for which the technology’s performance remains within that accuracy threshold. BioMeT community verification standards are typically covered by the IEC 60601 series of technical standards for the safety and essential performance of medical electrical equipment^21^. The series consists of collateral (IEC 60601-1-X) and particular (IEC 60601-2-X) standards. The latter define verification requirements for specific sensor signals. For instance, IEC 60601-2-26 specifies verification requirements for amplifier and signal quality properties for electroencephalography (EEG) sensors. IEC 60601-2-40 specifies similar criteria for electromyography sensors, IEC 60601-2-25 for ECG sensors, and IEC 60601-2-47 even focuses on requirements for ambulatory ECG sensors. Beyond these biopotential signals, specific standards do not exist for other commonly used sensor signals in BioMeTs (e.g., inertial, bioimpedance, and optical), leaving the definition of the verification criteria up to the manufacturer and regulatory authorities.

One challenge with establishing standard performance metrics is that performance requirements can vary by use case, and therefore the same technology performance may be sufficient for one scenario but not for another. For example, heart rate accuracy is critical for detection of atrial fibrillation in high-risk patients, but is less critical for longitudinal resting heart rate monitoring in healthy young athletes. The verification process, therefore, must include the intended use for designating appropriate thresholding criteria.

Verification serves as the initial step in a process in which data collected from further studies using the sensor technology are used to continue development of rational standards for use, uncover any unexpected sources of error, and optimize performance of BioMeTs.

## Who is responsible for verification?

Verification of BioMeTs is generally performed by the manufacturer through bench-top testing. Verification tests require access to the individual hardware components and the firmware used to process the sample-level data, both of which may be proprietary; as such, in some cases it may be impractical to expect anyone other than the technology manufacturer to complete verification. Indeed, many clinical investigators utilizing the technology will not have the resources or expertize required to perform such evaluations. However, it is likely the clinical investigators who will need to define the parameters of verification that would allow a determination of whether the sensor is, indeed, fit for a particular purpose.

Technology manufacturers should provide researchers and clinical users of their tools with timely and detailed verification documentation that is easily understandable to non-technologists. This documentation should be similar to the data sheets provided for hardware components, such as individual sensors that comprise the BioMeT. The documentation of BioMeTs should include three sections: performance specifications for the integrated hardware, output data specifications, and software system tests.

Performance specifications for the integrated hardware will mimic the performance specifications for individual hardware components but the testing must be completed on the full hardware system in situ. As an example, take a simple step counting BioMeT consisting of an accelerometer sensor and associated hardware to display the current daily step count on a small screen. Verification tests for integrated hardware performance specifications could include power management (expected battery life under a variety of conditions), fatigue testing (expected lifespan of the hardware under typical and extreme use), and/or electrical conductance (expected electrical current through the BioMeT).

Output data specifications should describe the accuracy of the sample-level data produced by the BioMeT’s sensors that will be used as input to the processing algorithms to produce the processed data. These verification tests usually consist of bench-top tests. These tests are necessary even if sample-level data are passed directly to the algorithms because, at a minimum, an analog to digital conversion of the sensor data may occur within the BioMeT. In the previous example of a simple step counting BioMeT, there is only one algorithm output metric: step counts. The sample-level data that are used as an input into that algorithm are the measurements that come from the on-board accelerometer as measured in SI units. The output data specifications should detail the accuracy of the accelerometer data in each axis (e.g., ± 0.02 g) as determined through bench-top testing of the full system, not just the accelerometer sensor.

Software system tests should indicate that the entire system including software that generates the sample-level data are functioning as intended, even under unusual circumstances of use. The results of the system tests do not need to be described in exhaustive detail in the documentation; instead, a high-level description of the software system tests should be included for general knowledge. For the step counter, this could include testing to ensure that the current step count is always displayed on the screen and is incremented within 1 s of a step being detected. An unusual situation would be to test what happens when the number of steps is so great that the size of the displayed digits exceeds the size of the screen (e.g., 100,000 steps per day or more). Other system tests could include what happens when the software detects an error within the system, such as a sensor malfunction.

Overall, the verification documentation for a BioMeT should give the clinical user enough information to use the BioMeT exactly as it was designed.

## What is the regulatory oversight of verification?

Regulation of verification testing in medical devices is currently overseen by the FDA in the US and the various Notified Bodies that conduct conformity assessments for CE marking in the EU^22^. These entities require specific verification testing before a medical device can receive clearance or approval. However, many BioMeTs are not required to go through the regulatory clearance/approval process, so independent verification standards for BioMeTs need to be developed.

There is a need for “verification standards” for BioMeTs that parallels the quality standards used to evaluate components of pharmaceuticals. In drug development, the United States Pharmacopeia^23^ is a non-profit organization that develops public guidelines for drug quality in collaboration with regulatory agencies, industry partners, and academia. An analogous organization for BioMeTs would be responsible for creating and updating guidelines and standards for verification testing. At present, there are multiple working groups within larger organizations that are focused on developing these verification standards for specific subsets of BioMeTs. Two examples of these working groups are the IEEE-WAMIII (Wearables and Medical IOT Interoperability & Intelligence) and the Consumer Technology Association’s Health and Fitness Technology Division. Such groups should collaborate to develop unified standards for verification that can be used by the regulatory bodies for oversight.

Table 3 describes the application of verification in practice.

**Table 3:** Verification in practice.

Documentation you can expect	Manufacturer should provide evidence of their BioMeT’s: • Performance specifications for the integrated hardware • Output data specifications • Overview of software system tests • Limitations to the verification testing • e.g., specific known items that were not tested during verification
Clinical users’ questions answered by verification	Is the performance of this BioMeT and each of its components sufficient to generate sample-level data of acceptable quality such that it can be used as an input to generate the processed data and downstream clinical measurement that I am interested in?

## Analytical validation

Analytical validation involves evaluation of a BioMeT for generating physiological- and behavioral metrics. This involves evaluation of the processed data and requires testing with human subjects^24^. After verified sample-level data have been generated by a BioMeT, algorithms are applied to these data in order to create behaviorally or physiologically meaningful metrics, such as estimated sleep time, oxygen saturation, heart rate variability, or gait velocity.

This process begins at the point at which verified output data (sample-level data), becomes the data input for algorithmic processing. Therefore, the first step of analytical validation requires a defined data capture protocol and a specified test subject population. For example, to develop an algorithm for gait velocity using data captured from a verified inertial measurement unit (IMU), it is necessary to specify (1) where the technology is worn (e.g., on the waist at the lumbar spine, ankle, or dominant wrist) and the orientation of the sensor, and (2) the study participant population (e.g., healthy adults aged 18–64, or patients with a diagnosis of multiple sclerosis aged 5–18)^25,26^. In this example, the analytical validation consists of evaluating the performance of the gait velocity algorithm on verified IMU data captured in accordance with the specific study protocol and in the particular study population of healthy adults aged 18–64.

During the process of analytical validation, the metric produced by the algorithm must be evaluated against an appropriate reference standard. Sleep onset/wake, for example, should be validated against polysomnography; oxygen saturation against arterial blood samples; heart rate variability against electrocardiography; and biomechanics such as gait dynamics against motion capture systems. It is important to remember that there can be multiple reference standards for a single metric, and not all reference standards are based on sensors. For example, a commonly used reference standard for respiratory rate is a manual measurement: a nurse observes and counts a study participant’s chest raises over a defined period of time. Manual reference standards are necessary when it is infeasible or impractical to use a sensor-based standard; step counts, for example, are typically validated using manual step counting rather than an instrumented walkway or instrumented shoes because it is more practical to have a human observer manually count the subject’s steps during a long walk test. In general, however, manual measurements are not the best choice for reference standards as they are the most prone to user error; they should only be used when absolutely necessary and no other reference standards are suitable and/or feasible.

It would be counterproductive to recommend a single threshold of accuracy for analytical validation of a BioMeT metric versus a reference standard as not all reference standards are of equal quality. First, not all reference standards are completely objective. For example, polysomnography signals are collected via sensors but may be manually scored by a trained technologist to generate sleep variables. Second, ostensibly objective reference standards like optical motion capture systems may have substantial operator bias that increases the variability of the final measurements^27^. Finally, in some cases a “gold standard” reference standard may not be clearly defined. For example, Godfrey et al. noted that the validation process for metrics produced by a gait algorithm based on body worn inertial sensors compared with a traditional laboratory reference standard, an instrumented pressure sensor gait mat, revealed poor agreement for variability and asymmetry estimates of left/right step data. In this case, a gait mat is a poor choice of reference standard to evaluate body worn sensors due to fundamental differences in measurement methods between the pressure and inertial sensor modalities^28^. Therefore, we recommend caution in the choice of reference standards for analytical validation studies. Most importantly, it is critical to understand how the selected reference standard measures and interprets the desired metric in order to undertake appropriate analytical validation procedures.

Best practices should be followed when choosing a reference standard for analytical validation of a BioMeT. The most rigorous and quantitative reference standards should be agreed upon and documented by guidance documents and consensus statements from governance and professional organizations. These are the reference standards that should be selected in order to avoid poor methodological approaches. Low-quality reference standards have the potential to introduce error as they may only produce an estimate of the desired metric. For example, a sleep diary contains the subject’s recollection of their sleep onset/wake time, which might vary considerably from the actual sleep onset/wake. Similarly, the process of back-validation, where analytical validation of a next generation BioMeT is evaluated against the previous generation, will also introduce error that can quickly compound if this process is repeated over multiple generations.

Table 4 summarizes the process of analytical validation.

**Table 4:** Summary of analytical validation.

Who?	Engineers, data scientists/analysts/statisticians, physiologists, behavioral scientists, and clinical researchers
What?	Protocol for data capture from a human participant. Algorithms applied to sample-level data to yield measurements that are indicative of clinical concepts.
When?	First use in human subjects.
Where?	Research or clinical laboratories.
Why?	To evaluate the performance of the algorithm, and its ability to measure, detect, or predict physiological or behavioral metrics.

## How can we reconcile analytical validation of BioMeT-generated measures in digital medicine with more familiar approaches from other disciplines?

BioMeTs come in a wide variety of form factors and levels of complexity. Despite this variation, the goals and challenges of generating evidence of analytical validity are common across many tools and are similar to those of non-digital tools. For example, both assessing the analytical validity of heart rate variability (HRV) from a commercial chest strap and gait velocity from a wrist-worn accelerometer require the use of reference standards, testing protocols, and statistical analyses that are widely accepted by subject matter experts. These elements have been a part of analytical validation within engineering and health-related disciplines for many years. However, questions of their relevance to BioMeTs of ever-increasing novelty can arise, particularly when the reference standards, testing protocols, and statistical analyses are poorly defined, non- intuitive, or are not disclosed at all.

In some instances, a BioMeT may be attempting to replace a less-robust clinical measurement tool that provides only measurement estimates (i.e., patient diaries). When it is not possible to robustly establish analytical validation due to the novelty of the data type generated from a BioMeT (i.e., no reference standard exists), then the need for evidence of clinical validity and utility increases. In contrast, the primary element required to demonstrate clinical validity (discussed below) is a reproducible association with a clinical outcome of interest. Methodological approaches to establishing associations are diverse and the most appropriate methods are dependent on the target population and context of clinical care.

## Who is responsible for analytical validation?

Analytical validation focuses on the performance of the algorithm and its ability to measure, detect, or predict the presence or absence of a phenotype or health state and must involve assessment of the BioMeT on human participants. As such, the entity that is developing the algorithm is responsible for analytical validation. Ideally, analytical validation would benefit from collaboration between the engineering team responsible for developing the sensor technology, data scientists/analysts/statisticians, physiologists or behavioral scientists, and the clinical teams responsible for testing in human participants from which the data are captured and the algorithm is derived. These multi-disciplinary teams might all sit within a single organization or may be split between a technology manufacturer and an analytics company, academic organization, and/or medical product manufacturer.

Commercial technology manufacturers often focus on developing generic algorithms with broad applications to a wide variety of subject populations in order to market their products to the widest possible consumer base. These algorithms (step count, walking speed, heart rate and heart rate variability, falls, sleep, muscle activation, etc.) could be applied to subjects with a variety of health conditions and under a variety of circumstances. However, commercial technology manufacturers may only conduct analytical validation for their algorithms using a small cohort of healthy subjects in a controlled laboratory setting. The manufacturer may or may not document the results of these studies in order to demonstrate the analytical validation of all the algorithms in their product. Sponsors of new medical products (drugs, biologics, or devices) choosing to use commercial technology will typically need to conduct their own analytical (and then clinical) validation.

When sponsors of new medical products (drugs, biologics, or devices) want to use BioMeTs to assess safety or efficacy of a new medical product for regulatory approval, they necessarily focus on developing specific algorithms with narrow applications that are targeted to their exact patient population of interest (e.g., Parkinson’s disease, multiple sclerosis, Duchenne’s muscular dystrophy). Through their clinical trial populations, sponsors generally have access to large data sets of patients with the specific health condition of interest from which to develop their algorithms. The trial sponsors may include a BioMeT prospectively as an exploratory measure in a clinical trial (both early and late stage) and use the collected data to develop the algorithm. There may be no available reference standards for these targeted algorithms; as a result, the sponsor may use other data collected during the clinical trial as the surrogate reference standards for the algorithms.

The sponsor should thoroughly document the analytical validation of the algorithms and is required to submit these results to regulatory bodies such as FDA or EMA. However, owing to the sensitivity of data collected during a clinical trial, these results may never be published or may be published years after the clinical trial has concluded. To demonstrate the efficacy of the BioMeT, we recommend that sponsors publish the results of analytical validation as soon as possible.

Table 5 describes the application of analytical validation in practice.

**Table 5:** Analytic validation in practice.

Documentation you can expect	Description of analytical validation studies conducted according to the requirements of Good Clinical Practice (GCP). This description can be in any one or more of the following forms: • Internal documentation • Regulatory submission (510 k) • White paper • Published journal article In the documentation, the evidence for every algorithmic output in their system: • Description of the output metric • Overview of how the metric was calculated, including specific details where possible • Which reference standard was used as the comparator to validate the metric • Results from a direct comparison between calculated metric and reference standard, including statistical analysis methods • Description of the human subjects population and experimental conditions and protocol used in the aforementioned direct comparison testing If this validation testing was undertaken as part of a clinical trial with human subjects, then the Institutional Review Boards (IRBs) or Ethics Committees (ECs) documentation should also be provided.
Clinical users’ questions answered by analytical validation	Can an algorithm acceptably measure, detect, or predict the presence or absence of a phenotype or clinical condition when that algorithm is applied to sample-level data captured by a verified sensor in accordance with a specific data collection protocol in a particular population?

## Clinical validation

Clinical validation is the process that evaluates whether the BioMeT acceptably identifies, measures, or predicts a meaningful clinical, biological, physical, functional state, or experience in the specified context of use. An understanding of what level of accuracy, precision, and reliability is necessary for a tool to be useful in a specific clinical research setting is necessary to meaningfully interpret results.

Clinical validation is intended to take a measurement that has undergone verification and analytical validation steps and evaluate whether it can answer a specific clinical question. This may involve assessment or prognosis of a certain clinical condition. Clinical validation should always be tailored to a specific context of use. The goal of clinical validation is to evaluate the association between a BioMeT-derived measurement and a clinical condition. The process of clinical validation also ensures the absence of systemic biases and can uncover BioMeT limitations such as an improper dynamic range to address a particular question. For example, a clinical validation could be determined in a study assessing the relationship between ambulatory BP monitoring and all-cause and cardiovascular mortality^29^.

Developing a standardized framework for clinical validation is challenging because of the highly variable nature of questions asked of clinical validation studies. However, we can adapt solutions from the FDA Guidance on patient reported outcomes^30^ or the CTTI recommendations and resources for novel endpoint development^31^. Some of the concepts such as defining meaningful change to interpret treatment response and ability to detect clinically meaningful change could be leveraged more extensively for the purposes of clinical validation for BioMeTs.

Clinical experts, regulators, and psychometricians who are experienced with the development of clinical measurement tools are intimately familiar with the process of clinical validation. The work that these experts do, does not change when the tool is digital.

Table 6 summarizes the process of clinical validation.

**Table 6:** Summary of clinical validation.

Who?	Clinical teams planning to use and generate scientific evidence based on the BioMeT in a stated context of use (which includes specifying the patient population).
What?	Well-designed clinical study protocols with appropriate inclusion/exclusion criteria, measurements, and outcomes to ensure assessment of content validity.
When?	After both verification of the data generated by the BioMeT and analytical validation of the data collection protocol and data processing by software algorithms is complete.
Where?	In the environment where the digital tool will be used. This will likely include data captured outside of the clinical or research laboratory environment during participants’ activities of daily living.
Why?	To evaluate whether the BioMeT acceptably identifies, measures, or predicts a meaningful clinical, biological, physical, functional state, or experience in the specified (1) population and (2) context of use.

## How can we reconcile clinical validation of sensor-generated measures in digital medicine with more familiar approaches from other disciplines?

Clinical validation is a process that is largely unique to the development of tests, tools, or measurements either as medical products themselves, or to support safety and/or efficacy claims during the development of new medical products, or new applications of existing medical products. Technology manufacturers who are not yet experienced in the clinical field may be unfamiliar with this final step in the development of a BioMeT. Equally, clinical experts with significant experience developing traditional clinical tests, tools, and measurement instruments may not realize that this process does not vary when developing and evaluating a BioMeT.

## Who is responsible for clinical validation?

Clinical validation is conducted by clinical teams planning to use, or promote the use of, the BioMeT in a certain patient population for a specific purpose. In practice, sponsors of new medical products (drugs, biologics, or devices) or clinical researchers will be the primary entities conducting clinical validation. If the digital tool is being used to support a labeling claim in the development of a new medical product, or a new application of an existing medical product, then the sponsor of the necessary clinical trials will be required to conduct clinical validation of any BioMeTs they use to make labeling claims.

In circumstances where the sponsor has completed analytical validation of an algorithm for a specific and narrow patient population, it may be possible to reuse some of the patient data that informed analytical validation to complete clinical validation. Clinical trials (both early and late stage) generate large data sets of patient health data that have traditionally been used to demonstrate clinical validity of biomarkers or surrogate endpoints^5^. This same process still applies when evaluating BioMeTs. We recommend using caution to avoid overestimating the utility of a digital endpoint if the same data set is used for both analytical and clinical validation. Documentation of clinical validation for BioMeTs should follow the same processes and requirements of clinical validation of traditional tests, tools, and measurement instruments^32^.

Table 7 describes the application of clinical validation in practice.

**Table 7:** Clinical validation in practice.

Documentation you can expect	Documentation of studies should include one or more of: • Clinical study report (CSR) • Regulatory submission (FDA or EMA) • White paper • Published conference proceeding • Published journal article Protocols and study reports should also be made publicly available. The Institutional Review Boards (IRBs) or Ethics Committees (ECs) documentation for the study should also be provided.
Questions answered by clinical validation	Can a BioMeT-derived measurement that has undergone verification and analytical validation steps be used to answer a specific clinical question?

## What is the regulatory oversight of the analytical and clinical validation processes?

The pathways for regulatory oversight of the validation processes will vary with the claims that the manufacturer of the BioMeT makes. For BioMeTs on regulatory pathways that require clearance or approval as a medical device, the centers within regulatory bodies responsible for these devices have regulatory oversight. These pathways are described in detail in Digital Medicine: A Primer on Measurement^3^.

For BioMeTs being used to support safety and efficacy claims of other medical products, there are a number of different options. In the United States, there is a pathway to “qualify” a digital tool outside of an individual drug development program^32^. Other pathways are specific to the medical product of interest. Decisions about the best approach to developing and/or a BioMeT in clinical trials and the preferred approaches for analytical validation should be made with input from regulatory agencies. CTTI has developed a quick reference guide to engage with the FDA for these conversations^33^.

## Real-world examples of V3 processes

Table 8 describes the application of V3 processes for five use cases, including both commercial and medical BioMeTs.

**Table 8:** Questions that verification, analytic validation, and clinical validation answer in example use cases.

Example use cases	Questions VERIFICATION answer:	Questions ANALYTICAL VALIDATION answer:	Questions CLINICAL VALIDATION answer:
Heart rate variability (HRV) from a commercial chest strap	Is the raw data from the ECG sensor on the commercial chest strap accurate, precise, and consistent? Are the processed RR intervals from the ECG sensor and post-processing on-board algorithms accurate with low errors^45^?	Does the HRV measured from the commercial chest strap ECG sensor provide clinical-grade accuracy of HRV (compared with a traditional ECG and Kubios clinical-grade software^45^?) Does HRV from the commercial chest strap meet standards set by the HRV Task Force^46^? Does HRV analysis meet the needs of users using the commercial chest strap (high accuracy under daily activities and during movement)^47^?	Can heart rate variability identify the presence of autism spectrum disorder in 8-year-old children^48^?
Gait speed from a commercial accelerometer	Is the accelerometer sensor accurate and precise within predetermined uncertainty? Is the accelerometer sensor raw data uniform and consistent?	Do the accelerometer sensor and processing algorithms provide clinical-grade accuracy of gait speed (compared to clinical automatic timing system used for gait speed analysis^49^ under the specific use case the device was developed for)^50^?	Can gait speed predict the onset of dementia in older adult patients^51^?
Arrhythmia detection	Is the heart rate sensor (optical heart rate or ECG) accurate, precise, and consistent? Does the post-processing algorithm for arrhythmia detection provide high sensitivity and specificity with low errors?	Does the arrhythmia detector (sensor and algorithms) meet the standards set by the FDA Class II Special Controls Guidance Document: arrhythmia detector and Alarm^52^? Does the arrhythmia detector provide information consistent with clinical review of ECG^53^?	Does the product acceptably detect atrial fibrillation (AF) in adults?
Closed-loop continuous glucose monitor (CGM)/glucose pump systems	Is the CGM sensor accurate, precise, and consistent with low errors? Is the pump system accurate, precise, and consistent with low errors? Does the closed-loop feedback algorithm provide timely, accurate feedback from the CGM to the pump consistent with FDA Considerations for Closed-Loop Controlled Medical Devices^54^?	Does the closed-loop CGM/pump system provide similar accuracy when compared with the current standard (system with multiple devices and manual calibration throughout the day?^55^) Do the closed-loop system components (CGM, pump, and feedback algorithm) meet specifications set by the FDA Regulatory Considerations for Physiological Closed-Loop Controlled Medical Devices Used for Automated Critical Care^54^?	Does this hybrid closed-loop system acceptably monitor glucose and automatically adjust the delivery of long acting or basal insulin based on the user’s glucose reading in the pre-specified context of use and patient population^56^?
Cuffless blood pressure (CBP) monitoring	Is the sensor used for CBP monitoring accurate, precise, and consistent with low errors? Is the algorithm used for determining BP accurate, precise, and consistent with low errors?	Does CBP monitoring provide clinical-grade accuracy (when compared to a traditional cuff BP monitor)^57^? Does the CBP device meet the standards for wearable devices issued by the Institute of Electrical and Electronics Engineers (IEEE 1708–2014^57,58^) and AAMIA Advancing Safety in Health Technology (ANSI/AAMI/ISO 81060-2:2013)^59^?	Do parameters of in-clinic blood pressure monitoring still apply to ambulatory/remotely captured blood pressure when considering the use of blood pressure as a prognostic biomarker for cardiovascular outcomes^29^?

## The V3 framework in practice

There are a number of considerations that transcend the processes of verification and analytical validation, and clinical validation in the development of BioMeTs.

## Do these processes replace existing GxP processes?

No. Good ‘x’ practices (or GxP) are guidelines that apply to a particular field. For example, ‘x’ may be manufacturing (GMP) or laboratory (GLP). Good practice guidelines apply to products in regulated fields (e.g., pharmaceuticals and medical devices) and are intended to ensure that these products are safe and meet their intended use by complying with strict quality standards throughout the entire process of production. V3 processes should be applied to all BioMeTs used in digital medicine. Digital tools that are also cleared or approved as medical devices must also comply with applicable GxP guidelines.

## Emphasizing the importance of a study protocol during V3 evaluation

It is important to develop clear study protocols and reports prior to embarking on V3 exercises. For verification, documentation should stipulate the requirements/acceptance criteria, testing steps, procedures, timelines, and documentation of the experimental results with appropriate conclusions. Both analytical validation and clinical validation processes are subject to regulations applicable to human experimentation. Clinical study protocols are required with an approval of IRB/EC and regulatory agencies, as applicable.

For all V3 processes, keeping appropriate test/study protocols and reporting the results is critical as it serves multiple purposes: defining the objectives of the experiment, aligning all stakeholders involved, complying with applicable regulations, and providing tools for determining compliance. In addition, protocols and study reports are key tools for documenting scientific evidence needed to draw inferences on whether a technology is fit-for-purpose for the intended use and context of use.

## Considering upgrades to firmware and/or software

The requirements for V3 are determined by the intended use of the BioMeT. Therefore, if the hardware or software are changed, new verification and/or analytical validation studies are needed to provide updated documentation for the end user (e.g., the study sponsor using the BioMeT as a drug development tool). Fortunately, changes in hardware and firmware often have no negative effects on the sample-level data, but the manufacturer still needs to demonstrate that this is true and also whether there is a “backwards compatibility” with earlier models. This is important because if an engineering improvement in BioMeT firmware or hardware makes the new data incompatible with data collected from earlier versions, this “improvement” could be disastrous for longitudinal studies and meta analyses.

Software updates that include changes to the algorithm processing the sample-level data require analytical validation to be repeated. However, if the hardware and firmware are unchanged, it is not necessary to repeat verification and analytical validation can be conducted using pre-existing sample-level data.

There can be misperceptions of the implications of firmware and software updates, such as whether or not those trigger new reviews from regulators like the FDA. For instance, software manufacturers are able—and encouraged by the FDA—to patch known security vulnerabilities^34^. Notably, software manufacturers, and not the FDA, are responsible for 640 validation of software changes after the patch has been deployed^34^.

## Extending BioMeTs to new populations

If the BioMeT itself has not changed, it is not necessary to repeat existing verification studies. However, whether existing validation data can be generalized to a different patient population or clinical setting is also a matter for scientific judgment and may require additional analytical validation and clinical validation studies. For example, consider an algorithm that processes data from a hip-worn accelerometer to generate the number of steps per day that was originally developed using data collected from healthy college athletes. There may be published data demonstrating that the algorithm performs well when tested on similar populations, such as people who are slightly older or those who are generally fit and active. However, it is unlikely, that the algorithm will generate an accurate step count if applied to a person suffering from peripheral neuropathy or a gait disorder. Thus, it would be incorrect to assume that just because analytical validation testing has demonstrated good performance in one scenario that the algorithm is then validated for use in all scenarios.

## Extending V3 concepts to multimodal and composite digital measures

V3 processes extend to multimodal data and composite digital measures. Multimodal describes data captured from two or more unique measurement methods. For example, a combination of accelerometer and gyroscope data can be used to detect falls and sit-to-stand transitions^35,36^. Digital tools relying on multimodal data should have evidence of verification available for each sensor, and evidence of analytical validation and clinical validation for the measure itself. Composite digital measures combine several individual measures, often derived from different sensors, to reach a single interpretive readout. For example, combining digital assessments of heart rate, sleep and heart rate variability can render a composite measure of depression^37^. Another example may combine accelerometer, GPS, keyboard and voice data from a smartphone to give a composite measure of cognition^38^. In these cases, verification of all contributing sensors is required along with validation of both the individual measures and the combined composite measure.

## How much validation is “enough”?

It can be difficult to decide whether an analytical validation study has achieved its goal of determining that an algorithm correctly captures the behavioral or physiological measure of interest. If there is a clear and objective reference standard, then a numerical accuracy threshold can be set a priori, and the algorithm can be said to be sufficiently well validated if the results of the testing meet or exceed the threshold. A numerical accuracy threshold should be chosen based on the expected accuracy of the reference standard combined with a literature review of relevant research and comparable validation studies that indicate what would be clinically meaningful accuracy. For example, heart rate has a clear reference standard (multi-lead ECG) and there are many published analytic validation studies describing the accuracy of various heart rate measurement devices^39^.

When evaluating a novel metric where there is no clear reference standard, analytical validation becomes a more challenging task. In such cases, the first step is to determine what level of accuracy is necessary to be clinically meaningful in the expected user population. This can be determined by a literature review of previously published research combined with consultations of key opinion leaders in the field. Once an approximate accuracy threshold has been established, the best available reference standard should be chosen. The reference standard is often the measurement method used in clinical practice, and should be chosen based on the literature and in consultation with key opinion leaders. Then the analytical validation study can be completed. It is noteworthy that the absence of a clear reference standard necessarily requires the integration of elements of analytical and clinical validation to appropriately evaluate the measure. An example of this type of study is the measurement of tremor in patients with Parkinson’s disease. Tremor is usually assessed by visual observation of the patient, which is not a clear reference standard. In one study, a BioMeT’s measurement of Percent of Time that Tremor is Present in Parkinson’s patients was assessed against visual observation to generate an accuracy score^40^.

In general, it is not possible to set a blanket threshold for all types of statistical assessments of clinical validation, as these will differ depending on the clinical measurement, patient population, and context of use. For example, a BioMeT that is highly sensitive to detecting a disease may be valuable for the purposes of screening owing to the low false-negative rate, whereas a BioMeT that is highly specific may be of value for the purpose of diagnosis owing to the low false-positive rate. Second, determining that the endpoint generated by the BioMeT is clinically valid and of importance to understanding the functional status or quality of life of the target population is critical. This process relies on examining the totality of evidence related to the endpoint in question, and using that information to make a scientific judgment as to whether the endpoint is an appropriate measurement or diagnostic marker. For clinical validation, the best practice would be to publish all available testing and results (including the protocols), which will allow future users to choose the most appropriate BioMeT for their specific purpose (fit for purpose).

**Fig. 3:**
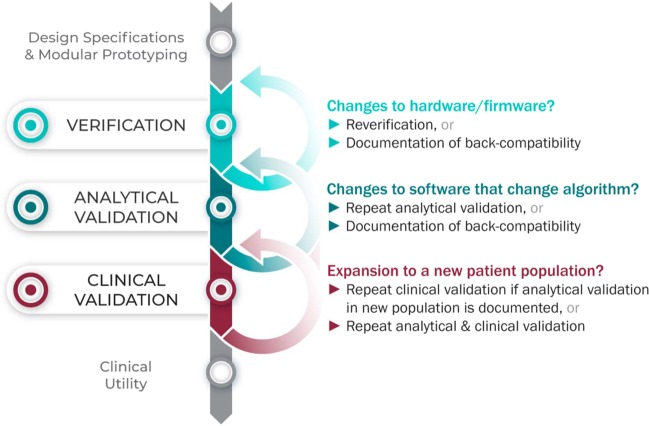
V3 in practice: The verification, analytical validation, and clinical validation process in the real world. The V3 process in practice.

Figure 3 summarizes the application of the V3 process in the real world.

## Statistical considerations in V3

Error can stem from a wide array of sources when employing BioMeTs. The development and implementation of a robust V3 protocol and subsequent BioMeT deployment and use in accordance with that V3 protocol will minimize error resulting from differences between expected and actual accuracy as well as intended and actual use. There are a wide range of statistical analyses used to evaluate BioMeTs for their coherence with reference standards and their clinical power, which is beyond the scope of this paper. Provision of raw data, whenever possible, helps to address transparency and independent evaluation of technologies by allowing independent investigation of, for example, data variance and its impact on BioMeT reliability. In addition, it is important to consider the limits of agreement if using different devices to quantify the same biomarker at different timepoints or in different cohorts.

## Future directions

Digital medicine is an interdisciplinary field, drawing together stakeholders with expertize in engineering, manufacturing, clinical science, data science, biostatistics, regulatory science, ethics, patient advocacy, and healthcare policy, to name a few. Although this diversity is undoubtedly valuable, it can lead to confusion regarding terminology and best practices in this nascent field. There are many instances, as we detail in this paper, where a single term is used by different groups to mean different things, as well as cases where multiple terms are used to describe what is essentially the same concept. Our intent is to clarify the core terminology and best practices for the evaluation of BioMeTs for use in clinical trials of new medical products, without unnecessarily introducing new terms. We aim for this common vocabulary to enable more effective communication and collaboration while improving the accessibility of the field to new adopters.

**Fig. 4:**
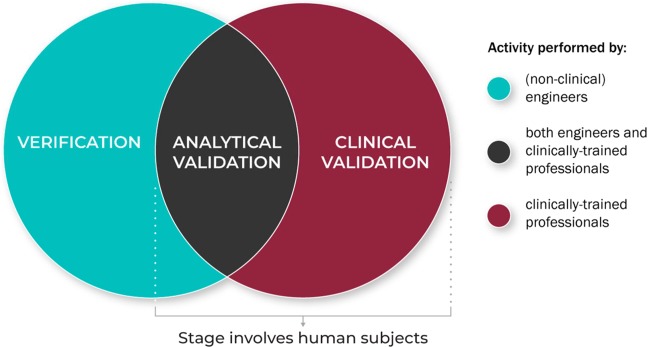
The role of the different disciplinary experts in the V3 process: Verification, analytical validation, and clinical validation processes are typically conducted by experts across disciplines and domains. V3 processes are typically conducted by experts across disciplines and domain.

Figure 4 summarizes the role of the different disciplinary experts in the V3 process.

V3 processes for traditional medical devices are generally well established but BioMeTs introduce new considerations^41^. For instance, SaMDs do not rely on specific hardware or sensors. The process of verification enables the use of SaMDs on verified data from any suitable sensor technology. In addition, some vendors sell “black box” algorithms or combined sensor/algorithm pairings. Establishing clear definitions and evidentiary expectations for the V3 processes will support collaborators seeking to evaluate the output of a “black box” sensor technology and/or measurement tool. Although the focus of this manuscript is on the use of BioMeTs in regulated trials of new medical products, our intent is for this framework to be instructional to all users of digital measurement tools, regardless of setting or intended use. Informing treatment decisions or care management based on a digital measure should not be subject to different scrutiny. Our goal in advancing this unifying V3 evaluation framework is to standardize the way high-quality digital measures of health are developed and implemented broadly. Evidence to support a determination of ‘fit-for-purpose’ and build trust in a digital measure should be uniform. A lack of V3 evaluation will have severe consequences (see Table 9 for illustrative examples) if algorithms fail to run according to predetermined specifications or if BioMeTs fail to perform according to their intended purpose.

**Table 9:** Illustrative examples of consequences where V3 evaluation does not occur.

Illustrative examples	Consequences
Cuffless blood pressure measurement	If the software for blood pressure estimation through a cuffless wearable was not carefully verified and validated, inaccurate blood pressure estimations used in clinical decisions may result in misdiagnosis and improper treatment that can result in patient harm.
Heart rate monitoring	Inaccurate heart rate monitoring could lead to improper conclusions about a patient’s risk for life-threatening cardiac events. Either over- or under-treatment in this scenario would likely result in patient harm and misallocation of health resources^60^.
Tapping on a smartphone to measure dementia	A BioMeT designed to detect dementia based on tapping patterns on a smartphone can diagnoses dementia in a healthy person if an older smartphone is used with a newer operating system because the delays and irregular tapping patterns are observed and misinterpreted by the BioMeT^61^. In this case, a carefully constructed verification process would have included testing the software in most, if not all, existing operating environment, so that the software specifications are met or the software usage is discouraged under certain conditions, and misdiagnosis owing to similar hardware system failures may be avoided. This example was witnessed firsthand by a member of our team.

Adopting streamlined methods for transparent reporting of V3 methodologies could lead to more ubiquitous deployment of low-cost technologies to better assess and monitor people outside of the clinic setting. This in turn can help healthcare professionals better diagnose, treat, and manage their patients, whereas promoting individualized approaches to medicine. Transparency will overcome “black box” technology development and evaluation approaches, ensuring that BioMeTs are used appropriately with the robust capture of data regardless of environment and context.

The proposed V3 process for BioMeTs describes an evidence base to drive the appropriate adoption of fit-for-purpose digital measurement technologies. In this document, we propose this three-pronged framework using historic and current contexts to define the key terms in this process. As a next step, we strongly encourage a re-initiation of the FDA B.E.S.T. working group to consider these definitions, refine them, and add them to the working compendium BEST framework^42^. We also encourage groups like the IEEE to consider these ontologies and provide feedback and guidance on the next steps required to adopt a common language and model for digital tools. We also recognize that technological developments will move faster than any regulatory or standards body can keep up with, so we encourage the practitioners in the digital era of medicine, including data scientists, engineers, clinicians and more, to continue to build upon this work. Professional societies like The Digital Medicine Society (DiMe) aim to become a collaborative hub for innovation in this area. Our hope is that the V3 framework and definitions continue to evolve to reflect the technologies that they serve. Our team will aim for annual updates to the framework as it exists herein. Once a common BioMeT evaluation paradigm is agreed upon, we will be able to develop technologies deserving of the trust we place in them (Boxes 1–3).

Box 1: Key takeaways

1. The term “clinically validated” is often found in marketing literature for digital medicine tools but, currently, its meaning is not clear. A standardized framework is needed to bring meaning to this term.
2. Biometric Monitoring Technologies (BioMeTs) are connected digital medicine tools that process data captured by mobile sensors using algorithms to generate measures of behavioral and/or physiological function.
3. The rapid rise in the demand for and development of digital medicine products, and specifically BioMeTs, to support the practice of medicine has left in its wake a body of new technologies with no systematic, evidence-based evaluation framework.
4. BioMeTs should be characterized by a body of evidence to support their quality, safety, and efficacy. Users of these technologies should recognize that verification and validation processes are critical to support a technology as fit-for-purpose. Without a supporting body of evidence, data can be misinterpreted. In the context of clinical trials, this can result in misleading study conclusions and possibly patient harm.
5. The evaluation framework for BioMeTs should encompass both the product’s components (e.g., hardware, firmware, and software, including algorithms) and the intended use of the product. Existing frameworks for new biotechnologies are not sufficiently adaptable, but they can provide meaningful insight for developing new evaluation frameworks for BioMeTs.
6. We propose and describe a three-component framework intended to provide a foundational evaluation of BioMeTs. This framework includes (1) verification, (2) analytical validation, and (3) clinical validation.
7. V3 are foundational to determine whether a digital medicine tool is fit-for-purpose. An evaluation of the usefulness and utility is only applicable after gaining evidence and assurance that the underlying data and predictions are “valid” to answer a given question.
8. Adopting streamlined methods for transparent reporting of V3 processes, coupled with transparency, will overcome “black box” technology development and evaluation approaches, ensuring that BioMeTs are used appropriately with the robust capture of data.

Box 2: Reality check—analytical validation in practice The process of conducting analytical validation as we describe it here is not always what happens in practice. Often algorithms are developed by technology manufacturers, are considered proprietary, and are not disclosed for testing. Sponsors of new medical products who want to use one of these tools to evaluate the safety or efficacy of a new product may therefore not have access to the algorithms. However, access to the algorithm itself is not necessary for the purposes of analytical validation, as long as the investigator is able to access the input data (sample-level data or processed data, depending on the algorithm) along with the software containing the algorithm in order to generate the endpoint/s of interest. Regardless of which party performs analytical validation, sponsors opting to use a particular BioMeT are responsible for their trial data integrity and communicating documentation of all stages of the V3 processes to regulators. Where IP issues prohibit sponsors from completing analytical validation independently, they must have means to assess analytical validation of the tools upon which their trial success depends.

Box 3: Sample-level and processed data Sample-level data are used as input to algorithms that convert that data to a second type of reported data that is not a direct representation of the original analog signal. We refer to this data as processed data because it is the result of processing operations applied to the original sample-level data. For example, ‘heart rate’ and ‘step count per minute’ are two processed data types that can be obtained from sample-level data (e.g., 250 Hz ECG or 50 Hz accelerometer, respectively).

In both cases, the processed data are not a direct representation of the original analog signal measured by the sensor; instead, an algorithm was applied to produce the new type of data. These processed data are almost always available to third parties and exists at a lower frequency than the sample-level data. In the case of sensor technologies that restrict access to the sample-level data, this processed data are the first-accessible data set from the device.

The distinction between sample-level and processed data are important because the evaluation processes differ. Following the V3 framework, sample-level data should be evaluated at the verification stage, and processed data should be evaluated at the analytical validation stage. This difference in evaluation processes is owing to the fact that the processed data have been manipulated from its original form.

## Supplementary information

Supplementary Information
