# Supplementary material for: Verification, analytical validation, and clinical validation (V3): the foundation of determining fit-for-purpose for Biometric Monitoring Technologies (BioMeTs)
Source: NPJ Digit Med. 2020 Apr 14;3:55. doi: 10.1038/s41746-020-0260-4 (PMC7156507; doi:10.1038/s41746-020-0260-4)
Supplement: Supplementary file 1 — Supplementary Information [file 41746_2020_260_MOESM1_ESM.pdf]

Supplemental Table 1: Key Definitions in Guidance Documents from Historical Frameworks

Source of Guidance	Guidance Objectives & Scope	Verification	Validation
**IEEE Standard for System, Software, and Hardware Verification and Validation (IEEE Std 1012™-2016)**<br><br>IEEE Computer Society<br><br>Sponsored by the Software and Systems Engineering Standards Committee<br><br>The Institute of Electrical and Electronics Engineers	This standard is a process standard that defines the verification and validation (V&V) processes in terms of specific activities and related tasks.<br><br>The purpose of this standard is to:<br>- Establish a common framework of the V&V processes, activities, and tasks in support of all system, software, and hardware life cycle processes.<br>- Define the V&V tasks, required inputs, and required outputs in each life cycle process.<br>- Identify the minimum V&V tasks corresponding to a four-level integrity schema.<br>- Define the content of the Verification and Validation Plan<br><br>The scope encompasses systems, software (including firmware and microcode), and hardware (an electronic or mechanical hardware element), and it includes their interfaces.	(A) The process of evaluating a system or component to determine whether the products of a given development phase satisfy the conditions imposed at the start of that phase. (B) The process of providing objective evidence that the system, software, or hardware and its associated products conform to requirements (e.g., for correctness, completeness, consistency, and accuracy) for all life cycle activities during each life cycle process (acquisition, supply, development, operation, and maintenance); satisfy standards, practices, and conventions during life cycle processes; and successfully complete each life cycle activity and satisfy all the criteria for initiating succeeding life cycle activities (i.e., builds the product correctly)..<br><br>Verification of interim work products is essential for proper understanding and assessment of the life cycle phase product(s).	(A) The process of evaluating a system or component during or at the end of the development process to determine whether it satisfies specified requirements. (B) The process of providing evidence that the system, software, or hardware and its associated products satisfy requirements allocated to it at the end of each life cycle activity, that is - solve the right problem (e.g., correctly model physical laws, implement business rules, and use the proper system assumptions), and satisfy intended use and user needs in the operational environment (i.e., builds the correct product).

**BEST (Biomarkers, EndpointS, and other Tools) Resource**<br><br>**FDA-NIH Biomarker Working Group, May 2018**	This document aims to capture distinctions between biomarkers and clinical assessments and to describe their distinct roles in biomedical research, clinical practice, and medical product development. The scope includes Diagnostic Biomarker, Monitoring Biomarker,Pharmacodynamic/Response Biomarker, Predictive Biomarker, Safety Biomarker, Susceptibility/Risk Biomarker, Prognostic versus Predictive Biomarker, and Surrogate Endpoints.<br><br>Adequate validation (e.g., analytical validation and clinical validation for biomarker tests) is important for applying a test, tool or instrument for any purpose, whether clinical or non-clinical, e.g., to elucidate disease etiology or pathophysiology, to predict clinical course (for use as a prognostic biomarker), to identify potential responders for predictive enrichment, or to represent clinical benefit (serve as a surrogate endpoint).	*Not Defined*	A process to establish that the performance of a test, tool, or instrument is acceptable for its intended purpose. Elements of validation include but are not limited to the following:<br>● **analytical validation** - A process to establish that the performance characteristics of a test, tool, or instrument are acceptable in terms of its sensitivity, specificity, accuracy, precision, and other relevant performance characteristics using a specified technical protocol (which may include specimen collection, handling and storage procedures. This is validation of the test's, tool's, or instrument's technical performance, but is not validation of the item's usefulness.<br>● **clinical validation** - A process to establish that the test, tool, or instrument acceptably identifies, measures, or predicts the concept of interest.<br><br>The following apply to clinical outcome assessments (COAs):<br>● **construct validatio**n: A process to establish, using quantitative methods, the extent to which the relationships among items, domains, and concepts of a clinical outcome assessment conform to a priori hypotheses concerning logical relationships that should exist with other measures or characteristics of patients and patient groups<br>● **content validation**: A process to establish from qualitative research the extent to which the clinical outcome assessment instrument measures the concept of interest including evidence that the items and domains of an instrument are appropriate and comprehensive relative to its intended measurement concept, population, and use.<br>● **criterion validation:** A process to establish the extent to which the scores of a clinical outcome assessment instrument are related to a known gold standard measure of the same concept. For most COAs criterion validity cannot be measured because there is no gold standard.

**CTTI, Mobile Technologies, July 2018**	This document is a guide for many considerations that accompany the decision to use mobile technology for data capture in clinical trials.<br><br>Recommendations are meant to provide a common framework that includes considerations of: Mobile Technology Selection; Data Collection, Analysis, and Interpretation; Data Management; Protocol Design and Execution; FDA Submission and Inspection<br><br>As one of the steps in selecting a mobile technology, its appropriateness should be justified through verification and validation processes.	The assessment of accuracy (which may include routine calibration), precision, consistency across time, uniformity across mobile technologies, and possibly also across different environment conditions. Verification also provides assurance that the relevant firmware /software that generates processed data is accurate, precise, consistent, and uniform.<br><br>Verification assures that the mobile technology reliably measures what it claims to measure, and is usually performed by the technology manufacturer with a series of engineering bench tests. This includes documentation about the technology's accuracy, reliability over time, and safety of the mobile technology and battery.<br><br>The measurement standard is usually a physical measurement such as acceleration, voltage, or time. The raw data are often processed within the mobile technology to deliver processed measures (i.e. acceleration data reported as activity counts).	The process of ensuring that the mobile technology is meeting its intended use by generating objective data that accurately represents the outcome assessment it purports to be measuring.<br><br>Validation assures that the processed data being assessed are suitable for its intended use and patient population in a trial.<br><br>If the ultimate measure of interest is not algorithm-dependent, validation may be synonymous with verification; however, if the ultimate measure of interest is algorithm-dependent, additional processes are required to validate the data generated by the mobile technology in the context of its use in the clinical trial.<br><br>Validation should occur in both a controlled environment (laboratory or clinic) and a real-world environment, and in the participant population of interest.

**Software as a Medical Device (SaMD): Clinical Evaluation, IMDRF, 2017 and 2015**<br><br>(SaMD = software as medical device)	This document, issued by the International Medical Device Regulatory Forum (IMDRF), provides a path for global regulators to converge on terminology, a risk-based framework, an understanding of quality management system principles, and an approach to making Software as a Medical Device (SaMD) clinically meaningful to users. V&V activities should be targeted towards the criticality and impact to patient safety of the SaMD.	Providing assurance that the design and development activity at each development stage conforms to the requirements.	Analytical validation measures the ability of a SaMD to accurately, reliably and precisely generate the intended technical output from the input data.<br><br>Clinical validation measures the ability of a SaMD to yield a clinically meaningful output associated to the target use of SaMD output in the target healthcare situation or condition identified in the SaMD definition statement. Clinically meaningful means the positive impact of a SaMD on the health of an individual or population, to be specified as meaningful, measurable, patient-relevant clinical outcome(s), including outcome(s) related to the function of the SaMD (e.g., diagnosis, treatment, prediction of risk, prediction of treatment response), or a positive impact on individual or public health. Clinical validation of a SaMD can also be viewed as the relationship between the verification and validation results of the SaMD algorithm and the clinical conditions of interest.<br><br>Valid clinical association, or scientific validity, refers to the extent to which the SaMD's output (concept, conclusion, measurements) is clinically accepted or well-founded (based on an established scientific framework or body of evidence), and corresponds accurately in the real world to the healthcare situation and condition identified in the SaMD definition statement.

**General Principles of Software Validation; Final Guidance for Industry and FDA Staff, 2002**	This guidance outlines general validation principles that the Food and Drug Administration (FDA) considers to be applicable to the validation of medical device software or the validation of software used to design, develop, or manufacture medical devices.<br><br>The Quality System regulation is harmonized with ISO 8402:1994, which treats “verification” and “validation” as separate and distinct terms. On the other hand, many software engineering journal articles and textbooks use the terms "verification" and "validation" interchangeably, or in some cases refer to software "verification, validation, and testing (VV&T)" as if it is a single concept, with no distinction among the three terms.<br><br>Software verification and validation are difficult because a developer cannot test forever, and it is hard to know how much evidence is enough. The level of confidence, and therefore the level of software validation, verification, and testing effort needed, will vary depending upon the safety risk (hazard) posed by the automated functions of the device.	Software verification provides objective evidence that the design outputs of a particular phase of the software development life cycle meet all of the specified requirements for that phase. Software verification looks for consistency, completeness, and correctness of the software and its supporting documentation, as it is being developed, and provides support for a subsequent conclusion that software is validated. Software testing is one of many verification activities intended to confirm that software development output meets its input requirements. Other verification activities include various static and dynamic analyses, code and document inspections, walkthroughs, and other techniques.	Software validation is confirmation by examination and provision of objective evidence that software specifications conform to user needs and intended uses, and that the particular requirements implemented through software can be consistently fulfilled. Since software is usually part of a larger hardware system, the validation of software typically includes evidence that all software requirements have been implemented correctly and completely and are traceable to system requirements. A conclusion that software is validated is highly dependent upon comprehensive software testing, inspections, analyses, and other verification tasks performed at each stage of the software development life cycle. Testing of device software functionality in a simulated use environment, and user site testing are typically included as components of an overall design validation program for a software automated device.

**NASEM 2017** [61]<br><br>**National Academies of Science Engineering & Medicine, 2017**	The National Academies of Sciences, Engineering, and Medicine convened a committee to examine the relevant medical and scientific literature to determine the evidence base for different types of genetic tests (e.g., predictive, diagnostic, and prognostic) for patient management. The committee provided recommendations to advance the development of an adequate evidence base for genetic tests to improve patient care and treatment. Additionally, the committee will recommend a framework to DoD for decision making regarding the use of genetic tests in clinical care.	Not Defined	Ideally, the clinical use of a genetic test should be preceded by studies to confirm that it is valid and useful. Two principal measures of validity apply to genetic tests: analytic validity and clinical validity. A third important measure of a genetic test is its clinical utility.<br><br>The **analytical validity** (technical test performance) of a genetic test is its ability to test accurately and reliably for the genetic variants of interest in the clinical laboratory in specimens that are representative of the population of interest. Analytic validity includes analytic sensitivity (false-negative results), analytic specificity (false-positive results), within- and between-laboratory precision, and assay robustness (reproducibility among operators, reagent lots, instruments, temperatures, and so on)<br><br>The **clinical validity** of a genetic test is its ability to identify or predict accurately and reliably the clinically defined disorder or phenotype of interest. Clinical validity encompasses clinical sensitivity and specificity and predictive values of positive and negative tests that take into account the prevalence of the disorder. Clinical validity might also be expressed as a measure of association, such as a risk ratio or an odds ratio, although such a measure is an incomplete representation of clinical validity.<br><br>The **clinical utility** of a genetic test is the evidence that it improves clinical outcomes measurably and that it adds value for patient management decision making compared with current management without genetic testing.

Supplemental Table 2

Equipment/ Technology	equivalence of a spring and mass (for example, Micro cantilever beam and a proof mass)	piezoelectric, piezoresistive or capacitive components	Analogue to digital converters	Wristband Microprocessor	Wristband On board computing	Bluetooth low energy Transmitter	Mobile Device Microprocessor	Wi-fi or Wireless Network	Cloud/Server-side processing
Resulting Signal	Deflection of proof mass from neutral position	Electrical Properties of Spring equivalent	Primary Digital Signal	1° processed digital signal	2° processed digital signal	2° Compressed, 2° processed digital signal	2° processed digital signal 2° processed digital signal	2° Compressed, 2° processed digital signal	3° processed digital signal
Information Produced (units)	Displacement (m)<br>Stress (N/m^2)<br>Strain (m/m)<br>And other properties	Capacitance (Farad)<br>Voltage (V)<br>Resistance (Ohm)	Digital representation of electrical properties	Acceleration in a single direction (m/s^2)	Steps / minute (counts)	Data Packets	Steps / minute (counts)	Data Packets	Daily steps (counts)
Information Storage Location	Equivalence of a spring (for example, the micro cantilever in MEMS)	piezoelectric, piezoresistive or capacitive components	Microprocessor input registers (or cache)	Wristband Flash Memory	Wristband Flash Memory	BLE Transmission buffer	Mobile device Flash Memory	Cloud or Server memory buffer	Cloud or Server Memory
